# Children with medical complexities: their distinct vulnerability in health systems’ Covid-19 response and their claims of justice in the recovery phase

**DOI:** 10.1007/s11019-022-10119-7

**Published:** 2022-11-16

**Authors:** Sapfo Lignou, Mark Sheehan

**Affiliations:** Ethox Centre and Wellcome Centre for Ethics and Humanities, Nuffield Department of Population Health, University of Oxford, Oxford, UK

**Keywords:** Covid-19, Children, Medical complexities, Vulnerabilities, Policy response, Fairness

## Abstract

In this paper, we discuss the lack of consideration given to children in the COVID-19 health systems policy response to the pandemic. We do this by focusing on the case of children with complex medical needs. We argue that, in broad terms, health systems policies that were implemented during the pandemic failed adequately to meet our obligations to both children generally and those with complex medical needs by failing to consider those needs and so to give them fair protection against harm and disadvantage. We argue that justice requires that the distinct needs and vulnerabilities of children with medical complexities are explicitly integrated and prioritised in decisions concerning healthcare and operational planning in the recovery phase and beyond.

## Introduction

When COVID-19 restrictions were first introduced in the UK in 2020, changes to healthcare provision were made across the board following the recommendations by professional bodies and commissioners to prioritise infection control and COVID-19 patient care (Hefferon et al. 2021). Adjusted healthcare delivery policies to respond to local COVID-19 challenges significantly affected the delivery of and access to child healthcare, in particular for children with long-term conditions and complex needs (RCPCH 2020). Restrictions to direct face-to-face clinician contacts, redeployment of staff from child health services and clinic space put non-urgent new referrals and key clinical activities on hold and markedly increased waiting lists experienced by community child health teams (Williams et al. 2021).

Non-communicable diseases represent the largest component of the burden of childhood diseases in the UK (Wolfe et al. 2013) but policy documents and ethical guidance issued (House of Commons Health and Social Care Committee 2020; NHS 2020; NHS 2021) made no specific provision for the care of children with chronic illness in their response to the pandemic. In our view this omission raises serious questions about the ability of the system to act justly when it comes to many children from this population group, and in particular those with medical complexities. While it is clear that these questions about policy making during the pandemic have not been acknowledged to date, there are also very serious questions about the extent to which they have ever been seriously considered. Our claims in what follows begins to address this gap. Drawing on both normative and empirical work, we distinguish important aspects of the particular vulnerability of this population group and discuss the ethical concerns which this omission in policy-making raises. We then offer a distinctive set of claims which locate and address the injustice in the processes of healthcare prioritisation and operational planning decisions in the recovery phase and beyond.

## The distinct vulnerabilities of children with complex medical needs

In what follows we distinguish a range of different kinds of vulnerabilities that children with complex conditions face. As we will see, these vulnerabilities were very much in play during the pandemic. There are a range of ways of classifying the vulnerabilities that we discuss below. ‘Interests’ is one plausible alternative. In our view ‘vulnerabilities’ captures the right relationship to harm in these cases: it is not that all people or all children suffer the harm to which they are vulnerable, nor do they suffer the harm in the same way or to the same extent. It seems reasonable to think that, in at least some cases, being vulnerable can itself bring about certain associated hardships (or harms) that are indirectly related to the vulnerability. (In this way we might begin to distinguish being at ‘risk of harm’ from being ‘vulnerable’.^1^)

Importantly, these vulnerabilities are all connected to broader obligations that fall on society in the form of its health system and on policy makers when they shape the institutions and behaviour of citizens in society. That these obligations exist is evident from the normal structures and institutions in our society that are designed precisely to deliver on these obligations of protection and welfare. If we turn to the ethics literature, we can also find robust argument for these obligations generally but also, specifically in the context of children and the obligations of society to protect, nurture and care for children.

So, let us take it that a person is vulnerable^2^, when they are at increased risk of harm from which they cannot sufficiently be protected, or they have a diminished capacity to meet their needs (Mackenzie et al. 2014; Gottfried et al. 2019). All human beings are to some degree vulnerable due to their fragility and dependency on others. However, children have certain characteristics that make them particularly vulnerable (Kottow 2004). Due to natural features of childhood like size, strength and cognitive capacity, and their lack of direct political, children are more prone to certain kinds of physical and emotional harm. For many of these harms, they have less ability to protect themselves than adults and must rely heavily on others, parents and carers, to be protected from them (Andresen 2014). Children with medical complexities or medically complex conditions have additional vulnerabilities due to the nature of their illness. They are prone to the symptoms of their condition, to pain and reduced quality of life and are in addition, highly dependent on healthcare and other institutional settings to meet their needs and help them function as close to normal as possible.

Children with medical complexities are diverse group and their vulnerabilities vary, among other things, depending on age, health condition, and developmental abilities. These vulnerabilities may diminish or increase as children grow up and develop their abilities and as their illness progresses. In what follows we refer to children with complex conditions or with medical complexities as those with either multiple healthcare conditions or with conditions which lead to multiple kinds of morbidities or comorbidities. In the former case, a child with autism spectrum disorder may also have diabetes or cancer. In the latter case, many rare childhood diseases like lysosomal storage disorders result in multiple system failures and require a range of health care interventions to manage each of the different sets of symptoms.

In our view it is helpful to disentangle this network of vulnerabilities so that we can understand their origin and become clearer about how policy makers should take each of them into account. We suggest four broad categories each of which overlap, intersect, and have difficult borderline cases. The first category of vulnerabilities captures the set of ‘frailties’ that are associated with being human generally. This is to be contrasted with the second category, those vulnerabilities that are associated with children and childhood specifically and in contrast to adults. We might say that this category is most broadly an age-related or ‘period of life’ set of vulnerabilities. Clearly other age groups have their own special vulnerabilities – old age being the clearest example – but since our interest here is with children, we force on those things which make children distinctively vulnerable. Unmet health needs in childhood can affect a child’s short and long-term status and function (Newacheck 2000) and become a defining feature of their adulthood by determining the additional threats to which a child will become vulnerable in the future (e.g., further disabilities and severe illnesses).

The next two categories that are relevant here are those related to complex and ‘non-complex’ conditions. Clearly anyone who suffers from an illness or disease of any sort is vulnerable to a range of illness-based harms. For our purposes here, this third category of vulnerability captures the set of harms that are attributable to a given condition. So, in simple terms, a broken leg with mean that the patient lacks mobility for a certain amount of time while the leg heals, is likely to have some level of pain to varying degrees through the healing process. There is also likely to be a range of broader consequences that could affect the patient in their broader lives: ongoing mobility issues (e.g. ability to exercise freely) or school, work or usual daily routines might be affected in the short or longer term.

We turn now to the final category that is of interest here. One important reason for separating complex conditions from other conditions is because in some cases the patient has a number of conditions (that may or not be related) or the condition itself has multiple different impacts. In both cases the range of vulnerabilities are exacerbated to not just include the individual affects but any mutually reinforcing influences. So, to take a simple case, patients suffering from both diabetes and cancer may be exposed to greater risks of side-effects when it comes to chemotherapeutic treatments.

Overall, the central reason for distinguishing each of these different categories of vulnerability is to help to exhibit the very distinctive position of children with complex conditions and to suggest that these children are at risk to an extent that is very much greater than each of the individual risks. Of course, children with complex conditions have each of the four categories of vulnerabilities. We suggest that these vulnerabilities are cumulative and interconnected rather than discrete and isolable. Protecting children from these cumulative and interconnected vulnerabilities require, as we have seen, multiple complex interventions and sophisticated packages of care that cannot be understood solely in terms of their individual components.

### Vulnerabilities and obligations

Acknowledging that children with medical complexities are vulnerable in distinctive ways is an important step towards being clear about our obligations towards them. These children are developing human beings and are significantly affected by both the nature of childhood and by the health condition or multiple health conditions from which they suffer. It seems reasonable to think that society generally has a *special obligation* that owed to all children *qua children*, and thus to children with complex conditions. This obligation might broadly be characterised as requiring appropriate protection from harms from such harms accordingly (O’ Neill 1998). This obligation does not only concern protection from active infringement of their bodily or mental integrity but also from *neglect* (Archard 2018). In the case of children with medical complexities these obligations require that their health needs are attended to and not ignored (Daniels 1981). Providing a child with the necessary treatment and protecting their physical and mental health integrity does not only serve their immediate interests as a child but also their developmental and future interests (Feinberg 1980). Obligations of care and concern are owed to children with long-term and complex medical conditions, as children, to provide them with best available services and goods (e.g. access to social and health related resources) to alleviate them from pain and suffering so that they can effectively operate in the world and become less vulnerable in their adult life (Kopelman 2012; Gottfried 2019).

Of course, the existence of the obligations to protect and care for children and the sick in our society does not give us much in the way of help when it comes to managing conflicts between these obligations and with questions about how to make policy which justly manages the competing vulnerabilities of those in society’s care.

### Pandemic policy and children with complex conditions

Broad-ranging, population-wide policy responses to the pandemic created problems for many patients who had their treatment being cancelled or postponed and who now must wait in a queue to receive care (BMA 2022). Yet, what makes children with medical complexities a *distinct* case for ethical consideration is the distinct vulnerability they faced due to the combination of their illness and their youth, or, more exactly, what is thought to be associated with the protection of their fragile health and developmental wellbeing. The impact on children with medical complexities when blanket service restrictions were introduced not only differed to that of other population groups that share some of their inherent characteristics but also increased the likelihood of those children to be more severely harmed or to bear additional types of harm in their actual and developmental wellbeing. Due to vulnerabilities related to their illness (their dependency on urgent and planned care and established therapeutic relationships) the health risks that many children with medical complexities were exposed to were greater than those faced by children with less health challenges were likely to face. Because they were children, children with medical complexities experienced additional types of harm associated with their developmental wellbeing and interests that do not normally threaten adults with same, complex health conditions.

On precisely this point, many health professionals and organisations expressed serious concerns in particular for the case of children with mental health, cerebral palsy and musculoskeletal problems who missed their developmental support and access to therapies since the start of the pandemic (Thorpe et al. 2021; Williams et al. 2020; Reilly et al. 2021). These problems were likely to be further exacerbated for some children with complex conditions facing other disadvantages such as unequal access to treatment (e.g. due to financial barriers in accessing remote care) and be compounded by other longstanding inequalities (e.g. poor health outcomes) (BMA 2021) As existing evidence has shown, due to language barriers and digital disparities, the care provided to some communities in the UK during the pandemic was diminished, impacting safeguarding procedures and widening health inequalities among paediatric patients (Watson et al. 2021; Horton 2018).

The adoption of a population-wide approach to service restrictions exposed an already vulnerable group to serious risks of harm by exploiting their inherent and situational vulnerabilities which, under normal circumstances, would not have been justified.

## What is a fair policy response to the pandemic fair for children with medical complexities?

Arriving at a fair pandemic response or a fair healthcare allocation policy during the pandemic necessarily involved assessing and weighing competing considerations. One of the hallmarks of justice in this context is that decisions regarding changes in healthcare provision or any of these related matters, need to be based on equal respect and concern for all (Larcher et al. 2020). The key to determining whether equal respect and concern has determined the policy rests with the process rather than the outcome. As we discuss below, by itself, the fact that one group did not receive adequate healthcare provision during the pandemic does not show that the policy in question was unjust. Instead, a lack of consideration of the needs of specific groups of patients combined with the lack of adequate healthcare provision resulting from a given policy show that the process was not robustly fair.

### Arguments about harms and benefits (on both sides)

The direct impact of COVID-19 infection on children’s physical health has been recorded as being far milder than for other groups but the indirect impact on their physical and mental health has been profound (Thorpe et al. 2021) Unmet health needs, delays to diagnosis for conditions that require swift diagnosis and treatment (e.g. cancer) (Maringe 2020) and the cause of avoidable deaths over the next five years due to missed appointments may suggest, as many health professionals have pointed out, that these children were unfairly used for the benefit of adults (RCPCH 2020).

According to the evidence, significant unmet need identified due to reduced access to healthcare and disruptions to planned outpatient visits or procedures could result in increases in morbidity and mortality for many children with various physical and mental health conditions (Thorpe 2021). In the case of children with developmental and epileptic encephalopathies for instance, it is estimated that some of them have suffered irreparable neurodevelopmental harm or premature morbidity as a consequence of delays in epilepsy surgery evaluations (Ogundele 2021).

Yet an argument suggesting that healthcare measures against the pandemic were unfair for children with medical complexities does not seem satisfactory when it ignores the impact of policies and measures on everyone else. When health services struggle with the viability of service provision because large numbers of staff are infected and timeliness is a key to the success of a health policy to contain the disease, changes across the board are necessary to reduce the spread of infection and protect provision. Though we might agree that we need to provide for the care of children with medical complexities, this does not mean that should be provided at all costs.

There are of course considerations that should be weighed against the potential harms to children. Though the direct risk of infection for children was low, the net benefit for some of them (e.g., those who were advised to shield) should not be neglected. Yet the number of the children that were likely to do worse if they contracted the virus was much smaller compared to that of adults for whom the threat of infection was imminent and thus a blanket approach for healthcare restrictions for the broader category of this population group cannot be easily justified.

Or again, children, and in particular those with medical fragility and intensive care needs, depend almost entirely on their adult carers, whose health could have been severely compromised if they contracted the virus. It can therefore be argued that although we are obliged to protect children with medical complexities from harm, there are situations where it not only is permissible but also essential to allow policies that diminish their well-being in order to promote a greater benefit, such as the reduction of the threat of infection from which they could derived indirect benefit.

### Justice as requiring equal consideration

These arguments show the difficulty of the policy-maker’s situation. The balance of considerations seems to justify the imposition of some risks on children’s physical and mental health for common benefit, but it does not address the question of how much is fair.

When assessing equalities and inequalities in the distribution of healthcare resources during the pandemic, it is clear that the immediate threat of the virus to some groups should have been prioritised over others, including the progressive illness of children with long-term conditions. During a pandemic outbreak, saving the most lives and prioritising the lives of those who are the sickest is intuitively preferable. As has been abundantly clear through the debates about lockdowns, testing and isolation, the extent to which those at the greatest risk through the pandemic, from the virus, should be protected at the cost to the health and welfare of others has been severely contested. In other areas of medicine, such as oncology and cardiology, the cost to patients of the dominance and prioritisation of those with COVID-19 has been clearly documented (Maringe 2020).

Our worry here however is twofold. First, consideration of less urgent needs, those that would have made some people progressively worse off, should have equally been taken into account. Though it may not have been possible to meet, either fully or adequately, the medical needs of children with medical complexities in times of crisis, our obligations to equally consider their needs in decisions that would affect their lives cannot be waived. Second, it is not simply that this group of patients were not considered, but that the processes themselves seemed unable to cope with the degree of complexity that children with complex conditions required for them to be fairly considered. In the former case, policy can be nuanced and can include acknowledgment of exceptions and mitigations. If complexity or children or children with complex conditions had been properly considered, blanket, population-level policies might not have been the norm. Efforts to mitigate these harms could have been made. These could have included the provision of alternative non-medical services, better information and psychological support for children and their families. In the latter case, generally applicable ethical frameworks and priority setting principles make no mention of how to manage complex cases and the policies that were produced did not specifically refer to children or complexity of care needs (House of Commons Health and Social Care Committee 2020; BMA 2021; Department of Health and Social Care 2020; NHS 2021; Wilkinson 2020; ). None of this is much of a surprise. The systems of priority setting and policy making in the healthcare system prides itself, as we have noted already, on equal respect and concern for all. This is often interpreted as “everyone counts as one and no one as more than one.” But as we have seen all things are not equal and the needs of some patient populations, in particular here, children with complex conditions are a long way from being equally vulnerable.

Issues of vulnerability and disadvantage require further reflection on the profound impact that policies have on the quality of our lives and prompt an evaluation of the ideological assumptions lying behind them. In previous and current healthcare policies and ethical frameworks (WHO/RFH/20.2; NHS 2020) the vulnerabilities of children with medical complexities are interpreted through an adult-centred perspective. Our ethical theories, particularly of distributive justice, seem not to consider children as a distinct group (Dwyer 2010). However, acknowledging children, and more specifically children with medical complexities, as deserving of care, respect and protection from harm, requires decision makers to attend to their distinct vulnerabilities and needs when considering actions that might affect them. By missing what is distinctively different about children with complex conditions as a group, including the severity of risks in which they were likely to be exposed to due to the fundamental role of healthcare provision in their actual and future wellbeing, implemented measures neglected the claim of those children to be treated in the government’s actions as equally morally considerable as those for who they aimed to protect against the high risk infection (CDC 2020).

## Ethical responses to children’s increased vulnerabilities in the recovery phase

The Covid-19 pandemic and its effects are expected to dominate healthcare delivery and access planning for at least the next few years (BMA 2020). As efforts, policy makers will continue to be forced to make difficult priority-setting decisions that will have a wide-ranging impact on different population and patient groups. In the immediate future, these decisions will need to both meet new care demands and reduce backlogs which resulted from the pandemic outbreak and continue to put pressure on resources. As acknowledged by the NHS guidance, (NHS 2021) such decisions should also aim to address health injustices by tackling inequalities in experience, access and health outcomes that compromised the health of those most vulnerable in the time of crisis.

The considerations of justice outlined above have important implications for these decisions going forward. We cannot categorically argue that health systems’ responses to covid-19 were unfair for children with complex conditions based only on their consequences. Though the impact on these children’s physical and mental health has been profound, some of the harms could not have been avoided or can even be outweighed by other benefits. What, however, we think makes health systems’ policy response unfair for children with medical complexities is the lack of consideration of their distinct needs and vulnerabilities when those decisions were made. Such injustice suggests interventions which aim to reduce and redress costs and burdens already imposed should also be considered.

Children with medical complexities should be given at least some healthcare priority in the recovery phase. Prioritising some patients based on differential evaluation of their vulnerability and the severity of harm incurred due to changes in healthcare provision is consistent with the NHS guidance (NHS 2021) which acknowledges that decisions regarding prioritisation should reflect different health needs and tackle health inequalities and injustices. Yet children with complex needs were neither the only vulnerable nor clearly the most vulnerable group subjected to severe harms during the pandemic. Let us for instance consider the impact of the healthcare restrictions on adults with terminal cancer, those with severe mental health challenges living in isolation or old people with multi-morbidities (BMA 2022). Assessing and suggesting the kind of priority that should be given to children with medical complexities in the recovery phase to compensate for these harms and injustices is therefore not straightforward. What nevertheless follows from our discussion, is that any selection of principles regarding prioritisation and resource allocation should take into account the inequalities and injustices resulting for this group in order to be fair.

The above argument may seem uncontroversial but unfortunately to date it has not been considered. The current NHS guidance for prioritisation and operational planning (NHS 2021) makes no specific provisions for the care of children with medical complexities in the recovery phase. Additionally, and in contrast to the NHS aim to address unfair health inequalities, it does not acknowledge that greater and more complex needs require greater resource focus and complex packages of care. These omissions further disadvantage this particularly vulnerable group while they seem to suggest that children with complex conditions waiting for treatment should always be deprioritised for the sake of efficiency or the maximisation of the number of patients treated.

Decisions that deprioritise the care of children with complex needs in the aftermath of the pandemic require active policy selection and justification. Lack of access to required healthcare services in both the pandemic and recovery phase blurs the distinction between actively imposed and retained risks as in effect many children waiting to receive treatment are exposed to the same level and type risk of harm. An account of just risk imposition implies a demand for fair sharing by all members of society by taking into account their morally relevant differences. Prima facie duties towards children with complex medical conditions should shape assumptions about what constitutes acceptable risks that can be impose to them for the benefit of others and shape decisions and priorities when obligations towards them cannot entirely be fulfilled. Attending to the concerns raised by children’s increased vulnerability and disadvantage requires taking steps to ensure that, at least in the recovery phase, prior disadvantage does not embed into poorly designed policies. We suggest that a revision of the current NHS criteria for healthcare prioritisation is warrant in order the needs of these children to be given proper weight in any deliberation about resource allocation.

The responsibility that lies with the state and health institutions to reduce preventable causes of corrosive vulnerability in children with chronic illness and to ensure that their needs are fairly met is not only relevant to the current waiting lists for treatment but also in addressing system previous insufficiencies affecting their care. Pre-pandemic problems, such as the increasing ineffectiveness of the hierarchical referral model of primary to secondary care (Horton 2018) and variable and sometimes poor outcomes among children with chronic illness, reflect the need for structural changes such as redesigning and improving their care pathways (RCPCH 2020) to ensure that systemic inequities and previous health inequalities are not sustained in the aftermath of the pandemic.

Finally, it is essential to examine the structural factors and background conditions that diminish or increase vulnerabilities and lead to disadvantages that go beyond clinical care (Wolff 2011) and which children and their families are not in a position to change effectively to improve their situation. These considerations are part of more central concerns about children’s position in society. Government actions, such as covid-19 and non-covid-19 related policies and practices, can positively or negatively affect the health and the quality of children’s lives and address the factors that promote their wellbeing. To enable children with chronic illness to escape from corrosive vulnerability in the aftermath of the pandemic and the different kinds of harm that would result from it, factors that create and sustain vulnerabilities and harm need to be critically evaluated and addressed. This suggests that different types of evidence are important (Lignou et al. 2022) to inform policies in addressing the longer-term impact of Covid-19 on child health and plans for future pandemics, and for identifying strategies that can eliminate systemic weaknesses in meeting the complex needs of children with chronic conditions more generally.

## Conclusion

Justice requires that inequities and prior disadvantage are addressed in current policies regarding the recovery of healthcare services and decisions about prioritisation. By recognising the importance of integrating the distinct needs and vulnerabilities of children with medical complexities into current approaches to healthcare prioritisation, it is hoped that this work will encourage further research on how the interests of population groups who lack direct political voice, including children with medical complexities, should be integrated into our moral thinking and inform health policy decisions in the recovery phase and beyond.

## Footnotes

1 Although it seems right to say that ‘being vulnerable’ and ‘having a certain risk’ are strictly very close, we might want to separate out these added components that are associated with vulnerability. So, we might think that all people have some risk of being injured by a random event at any given time, and in virtue of this, they are vulnerable to this kind of injury, we might want to distinguish this from being vulnerable in a fuller sense.

2 There are a whole set of issues associated with the concept of ‘vulnerability’ which are beyond the scope of this paper. The brief, schematic account which we suggest here is intended to be a placeholder to capture, in broad terms, the ordinary sense of the term and which we take to be uncontroversial.
